# Stacking Models of Growth: A Methodology for Predicting the Pace of Progress to the Education Sustainable Development Targets Using International Large-Scale Assessments

**DOI:** 10.1017/psy.2025.2

**Published:** 2025-02-13

**Authors:** David Kaplan, Kjorte Harra, Jonas Stampka, Nina Jude

**Affiliations:** 1 Educational Psychology, University of Wisconsin-Madison, Madison, WI, USA; 2 Institut für Bildungswissenschaft, Universität Heidelberg, Heidelberg, Baden-Württemberg, Germany

**Keywords:** Bayesian growth modeling, ensemble modeling, large-scale assessments

## Abstract

To assess country-level progress toward these educational goals it is important to monitor trends in educational outcomes over time. The purpose of this article is to demonstrate how optimally predictive growth models can be constructed to monitor the pace of progress at which countries are moving toward (or way from) the education sustainable development goals as specified by the United Nations. A number of growth curve models can be specified to estimate the pace of progress, however, choosing one model and using it for predictive purposes assumes that the chosen model is the one that generated the data, and this choice runs the risk of “over-confident inferences and decisions that are more risky than one thinks they are” (Hoeting et al., 1999). To mitigate this problem, we adapt and apply *Bayesian stacking* to form mixtures of predictive distributions from an ensemble of individual models specified to predict country-level pace of progress. We demonstrate Bayesian stacking using country-level data from the Program on International Student Assessment. Our results show that Bayesian stacking yields better predictive accuracy than any single model as measured by the Kullback–Leibler divergence. Issues of Bayesian model identification and estimation for growth models are also discussed.

In 2015, the Member States of the United Nations (UN) adopted the *2030 Agenda* often referred to as the *Sustainable Development Goals* (SDGs) (UN General Assembly, 2015). With regard to education, the UN identified equitable, high-quality education, including the achievement of literacy and numeracy for all youth as well as adults as one of its global SDGs to attain by 2030 (UN General Assembly, 2015). To assess country-level progress toward these global goals, it is necessary to monitor trends in educational outcomes over time. Clearly, trends in educational outcomes have been seriously interrupted by the COVID-19 global pandemic, and indeed, the recent release of the 2022 *Program for International Student Assessment* (PISA) results by the Organization for Economic Cooperation and Development (OECD) showed that between 2018 and 2022 average proficiency in mathematics dropped by almost 15 score points and almost 10 score points for reading, while science remained relatively stable from the 2018 results (OECD, 2023b). To put this into perspective, the results show that, at least for mathematics, students lost about three-quarters of a year of instruction (Avvisati & Givord, 2023). The OECD report recognizes that trends in mathematics and reading were declining prior to 2022, but that the drop observed in 2022 was vastly larger than any drop previously recorded. Thus, as educational systems around the world face unprecedented challenges due to the COVID-19 pandemic, monitoring trends in educational outcomes could help identify the long-run impact of this unprecedented health crisis on global education. To this end, international large-scale assessment (ILSA) programs such as the OECD’s PISA (PISA, OECD, 2001) are uniquely situated to provide population-level trend data on literacy and numeracy outcomes.

The purpose of this article is to demonstrate how optimally predictive models can be constructed in order to monitor the pace at which countries are moving toward (or away from) the education SDGs, and, importantly, to understand the impact of the pandemic on slowing down the pace of progress. A number of models can be specified to estimate the pace of progress, and these can include models for linear and non-linear trends, including time-varying and time-invariant predictors. However, choosing one model and using it for predictive purposes assumes that the chosen model is the one that generated the data. Choosing a single model runs the risk of “over-confident inferences and decisions that are more risky than one thinks they are” (Hoeting et al., 1999). To mitigate this problem, we adapt and apply *Bayesian stacking* (Breiman, 1996; Wolpert, 1992; Yao et al., 2018), a method for ensemble prediction that arose out of machine learning, to form weighted mixtures of predictive distributions from an ensemble of individual growth curve models, with weights based on leave-one-out log predictive density scores. Bayesian stacking is an improvement over the more classical approach of *Bayesian model averaging* (BMA) (e.g., Draper, 1995; Hoeting et al., 1999; Madigan & Raftery, 1994) insofar as BMA assumes that the correct data generating model is in the space of models being averaged. Bayesian stacking makes no such assumption, and in principle, should demonstrate better predictive skill than that of any single model chosen for predictive purposes.

The organization of this article is as follows. In the next section, we describe the current procedures used by the OECD and the UN to measure the distance to the SDG targets. Next, we provide our critique of the approach used by these agencies. Following, we outline the specification, identification, and estimation of growth curve models from a Bayesian perspective that sets the focus for our estimation of the pace at which countries are moving toward, or away, from their education SDG targets. We refer to this simply as the *pace of progress*, or *pace*. This is followed by a discussion of Bayesian probabilistic prediction as situated in Bayesian decision theory and in the context of estimating pace. Next, we discuss different *modeling frameworks* that set the stage for the assumptions underlying Bayesian ensemble prediction using Bayesian stacking. This is followed by our example of Bayesian stacking of models for the pace of progress using country-level data from PISA 2009–2022. On the basis of the optimal estimates of pace, we then provide an example of forecasting PISA results one-cycle ahead, namely for PISA 2025. The paper closes with the presentation of a possible workflow for estimating trends and providing predictions from a Bayesian perspective as well limitations of this article and the insights that can be gleaned from the predictive point of view applied to large-scale assessments of the education SDGs.

## 1 Current methodology for measuring distance to SDG targets

A key argument of this article is that the progress countries are making to achieve their SDG targets generally, and in education particularly, can be conceptualized as a rate of change. However, the major policy reports on country-level progress to the SDG targets presents the problem as one of measuring the *distance* to the target of interest. A review of the extant reports on measuring the distance to the SDG targets have revealed differences in approaches depending on whether the report emanates from the OECD or the UN. In this section, we review the approaches taken by both organizations along three lines (a) selection of indicators, (b) specification of target levels, (c) calculating distance measures, and (d) estimating trends. Our review of the methodologies used by the OECD is taken from OECD (2019). Our review of the methodologies used by the UN are taken from Lafortune et al. (2018)).

### 1.1 Selection of indicators

There are many ways to measure how far countries are from their 2030 targets. The procedures used by the OECD are as follows. First, suitable data sources must be identified. Where OECD data are aligned with the UN Global Indicator List, OECD data are used. Where no OECD data sources exist, data are then extracted from the UN Global Database. Finally, where neither OECD nor UN Global Database data are in full alignment with the UN Global Indicator List, then OECD data that are considered suitable as close proxies are used.

As with the OECD methodology for indicator selection, the UN reports indicators that have been adopted by the UN Statistical Commission. However, where data gaps are identified, the UN uses 5 criteria for indicator selection and inclusion (Lafortune, Fuller, Moreno, Schmidt-Traub & Kroll, 2018):

1. **Global relevance and applicability to a broad range of country settings**: The indicators are relevant for monitoring achievement of the SDGs and applicable to the entire continent. They are internationally comparable and allow for direct comparison of performance across countries. In particular, they allow for the definition of quantitative performance thresholds that signify SDG achievement.
2. **Statistical adequacy**: The indicators selected represent valid and reliable measures.
3. **Timeliness**: The indicators selected are up to date and published on a reasonably prompt schedule.
4. **Data quality**: Data series represent the best available measure for a specific issue, and derive from official national or international sources (e.g., national statistical offices or international organizations) or other reputable sources, such as peer-reviewed publications. No imputations of self-reported national estimates are included.
5. **Coverage**: Data have to be available for at least 80% of the 149 UN Member States with a national population greater than one million.

### 1.2 Specification of target levels

In the next step, target levels are required. The OECD selects target levels that are explicitly specified in the 2030 Agenda are used. However, when no target value is identified in the Agenda, target levels are drawn from other international agreements (e.g., based on World Health Organization targets). If no target value can be identified, then the target level is set to the current “best performance” among the OECD countries. If none of these can be found or used, then no target level is set and no distance is calculated.

For the UN, target levels are set according to the following steps:

1. Use absolute quantitative thresholds in SDGs and targets: e.g., zero poverty, universal school completion, universal access to water and sanitation, full gender equality. Some SDG Targets propose relative changes. For example, indicator 4.1.1 requires measuring the “Proportion of children and young people (a) in grades 2/3; (b) at the end of primary; and (c) at the end of lower secondary achieving at least a minimum proficiency level in (i) reading and (ii) mathematics, by sex.” This indicator presumes that a quantitative threshold for minimum proficiency exists and can be applied more or less universally. In this case, the minimum proficiency level in PISA (Level 2) (OECD, 2024) could serve this purpose, and it is implied that meeting the indicator 4.1.1 means that there would be no individuals below level 2 by 2030.
2. When no explicit SDG target is available, apply the principle of “leave no one behind” (https://unsdg.un.org/2030-agenda/universal-values/leave-no-one-behind) to set an upper bound to universal access or zero deprivation. This principle was applied to targets addressing measures of extreme poverty, public service coverage, and access to basic infrastructure.
3. When science-based targets exist that must be achieved by 2030 or later, use these to set 100% upper bound. Here again, PISA level two could, arguably, be considered a science-based indicator for 4.1.1 given the skills and competencies that scholars have defined as minimum competency in the domains of reading and mathematics. A bound on this indicator would mean that 0% of boys and girls are below minimum competencies.
4. When several countries already exceed an SDG target, use the average of the top five performers.
5. For all other indicators, use the average of the top performers. In the case of global indicators retained, the upper bound was set by taking the average value of the top five global performers. For OECD indicators, the average top three performers.

### 1.3 Calculating a distance metric

For the purposes of understanding the pace at which every country is progressing toward the target levels, there is a need for a common scale of comparison. For some of the SDGs, the target levels, and hence the calculation of distances, are relatively straightforward. For example, SDG Target 1.1 states “By 2030, eradicate extreme poverty for all people everywhere, currently measured as people living on less than $1.25 a da.” Although exact targets were available for 47 of the 132 indicators used for the education goals, somewhat more vague targets were specified making it difficult to provide a reliable distance metric. For example, indicator 4.1.1 specifies “Proportion of children and young people (a) in grades 2/3; (b) at the end of primary; and (c) at the end of lower secondary achieving at least a minimum proficiency level in (i) reading and (ii) mathematics, by sex,” and this leaves it to the countries to define what is meant by “minimum proficienc.”

To create a common metric, the OECD average distance is the population-weighted average of distances across all OECD countries using the population in 2016 as weights. A “standardized difference” is calculated as a z-score difference between a country’s current position and the target end-value (OECD, 2023b).

### 1.4 Measuring trends

A distance measure between the current status of a county on an SDG indicator and a set target is useful information, but clearly not sufficient to provide an understanding of progress toward the stated target. What is of interest is the measurement of trend toward the target. For the OECD report, a Spearman rank-order correlation between the observed values of each indicator and time (in years) is calculated. For example, if the trend is significantly below 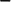 0.20, then the trend is interpreted as a “movement away from the SDG target.” If the trend is significantly above 0.20, then the trend is interpreted as “progress toward the SDG target.” In between them “no consistent trend could be identified.”

## 2 Critique of the OECD and UN methodologies

The importance of the OECD and UN reports notwithstanding, there are several problems with their methodology for computing the distance measures that need to be raised.

### 2.1 Missing data

In the OECD report, it is unclear how missing data is actually treated. The plots provided by the OECD show uncertainties based on two assumptions made regarding the missing data (a) that the missing indicators are all three standard deviations from the target level, and (b) that the target level has been achieved. In no place is there a discussion of the reasons that a country might be missing the indicators necessary to construct a distance measure. Without explicit treatment of the missing data, any number of biases can enter the analysis, and indeed, the OECD report recognizes this problem. The theoretical work on missing data is well developed, and the implications associated with why data are missing can directly impact the associated distance measures. In addition, it does not seem that there were attempts to statistically address missing data such as through imputation methods.

### 2.2 Measurement error

There is no discussion of measurement error in the indicators. A large number of indicators are indexes comprised of individual measures, and so the reliability of these measures could be calculated. In the case of Indicator 4.1.1, what is defined as “minimum proficiency” would be dependent on the assessment that is being employed. For assessments such as PISA, reliability information is readily available. In the case of lesser-known country-level assessments, reliability information needs to be provided. Information regarding reliability is particularly important insofar as it is well known that the validity of a measure cannot exceed the square root of its reliability, and hence measuring the relationship of target indicators to each other is directly impacted by the reliability of the measures involved.

### 2.3 Difference scores

The above issues notwithstanding, the calculation of the standardized difference between where a country is now and how far it is from the stated target cannot provide an assessment of the pace at which countries are moving toward their stated targets. First, the difference score does not indicate how a country got to where it is now. It only measures the linear difference between the target and current status. As such, the difference score does not (and cannot) pick up non-linearities over time in progress toward the targets because the past trajectory is not part of the calculation of the difference score. Second, the difference scores are calculated for each country and each indicator separately, and so does not borrow information from other countries. For some indicators, this might not be a serious problem, but for others, trans-boundary movement to the targets may be important and one would want to utilize all available information, including that of other countries, to estimate the pace at which a given country is moving toward the targets.

It seems that the OECD recognized these limitations. In fact, the OECD report states: “These results should, however, be interpreted carefully. Progress toward the target says nothing about whether the pace recently achieved by a country would be sufficient to meet the target level by 2030. The evidence … should therefore be considered as only a first step toward a more extensive analysis that would allow target-by-target projections of the future trajectories for each country.” (OECD, 2019, p. 34)

This article considers the limitations recognized by the OECD as vitally important for assessing progress toward the SDG education targets. For this article, we conceptualize the problem as one of assessing the pace at which countries are moving toward (or away) from the targets, and importantly, we conceptualize gender inequality as one of gender differences in the pace of progress. However, it is important to recognize the pervasive uncertainty inherent in specifying models to estimate the pace of progress, and so to this end, we develop an approach to assessing the pace of progress to the SDG targets based on Bayesian growth curve modeling and Bayesian ensemble prediction.

## 3 Bayesian growth curve modeling: Specification, identification, and estimation

In this section, we outline the specific form of the growth model that we will employ in our empirical example. In anticipation of our example, we will contextualize the problem as one of predicting the pace of progress in country-level mathematics proficiency as measured by PISA. To begin, we write the intra-country model of the pace of progress in mathematics outcomes as 
(1)

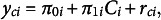

where 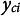 is mathematics outcome for country *i* ( 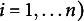 at PISA assessment cycle *c* ( 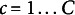 ), 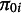 is the intercept capturing country *i*’s proficiency level at a fixed point in time (usually the first cycle—for this article, 2009), 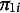 is the slope (linear pace of progress over time) in mathematics proficiency for country *i* at cycle *c*, and 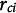 is the residual term.^1^ Together 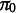 and 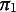 are random effects, typically referred to as *growth parameters* in the growth curve modeling literature (e.g., Bollen & Curran, 2006; Grimm et al., 2017). For this article, 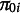 is the estimated 2009 country-level achievement for country *i*, which we will refer to simply as the *starting point*, and 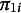 is an estimate of the *pace* at which country *i* is increasing or decreasing in mathematics proficiency. Our goal is to obtain optimal estimates of these parameters to be used to predict future mathematics proficiency scores. The linear growth curve model in Equation (1) can be extended to include time-varying predictors as 
(2)

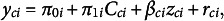

where 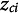 is a predictor that also changes over time with the outcome and is hypothesized to predict the outcome at cycle *c*, but is not an outcome of interest in itself. The parameter 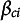 describes the size and sign of the prediction. An example of a time-varying predictor might be the number of math teachers in country *i* at cycle *c*.

It is well known that higher order terms can be specified, such as quadratic pace of progress, which can be written as 
(3)

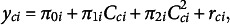

and indeed, the latent variable modeling approach to growth curve modeling allows for considerable flexibility in non-linear curve fitting (see Bollen & Curran, 2006; Grimm et al., 2017), and our approach to stacking growth models can be easily extended to allow the analyst to focus on any shape component (linear or non-linear) of substantive interest.

An important flexibility in growth curve modeling allows for the estimation of non-linear trajectories using *latent basis* methods. This specification requires that some of the time points be fixed to constant values while allowing the remaining time points to be estimated from the data directly. Latent basis modeling yields data-based estimates of the time points and often provides better fit of the model to the empirical trajectories of change over time compared to forcing, say, a linear trend on the data. In the context of latent basis methods, the pace parameter 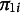 is best conceived of as a *shape* parameter, but we will continue to refer to this as a pace of progress parameter. These, and other extensions, are discussed in Bollen & Curran (2006) and Grimm et al. (2017). For this article, we will examine differences in predictive performance across three latent curve models: (a) a simple linear growth curve model, (b) a latent basis model with that last time point (2022) freed to reflect a decrease in scores due to the COVID-19 pandemic, and (c) a latent basis model in which the basis functions from 2015 to 2022 are freed, reflecting the idea that the trend downward had started prior to 2022.

In addition to adding time-varying predictors to the model as in Equation (2), it is common practice to model the starting point and pace parameters as a function of time-invariant predictors. For this article, the inter-country model for the starting point 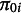 and pace 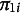 , respectively, can be written as 
(4)

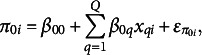

and 
(5)

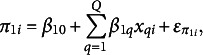

where 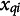 are values on *Q* predictors for country *i*, 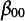 , 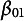 , 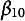 , and 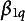 are the intercept and slopes associated with the time-invariant predictors of the starting point and pace of progress, and 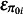 and 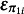 are errors. An example of a time-invariant predictor might be the type of political system of a country.^2^ For this article, we will develop a set of distinct models for predicting 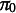 and 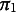 .^3^

The Bayesian framework for the growth curve model in Equations (1) through (5) requires specifying a probability model for the outcome and placing priors on all model parameters (Kaplan, 2023). Following Kaplan & Huang (2021), the priors for our growth curve models will be non-informative or weakly-informative (see, e.g., Gelman et al., 2017). This choice allows the data to “speak” while stabilizing the analysis without impacting inferences, particularly in the presence of small sample sizes (Kaplan, 2023).

### 3.1 Bayesian model identification

The issue of identification in the Bayesian context is somewhat different than what is commonly understood as identification in the frequentist framework (Fisher, 1976). The topic of parameter identification from the Bayesian perspective goes back at least as far as Lindley (1971) who remarked that identification was not really an issue in Bayesian inference because when a proper prior distribution is specified, the posterior will always exist. More recently, Florens & Simoni (2021) showed (among other things) that there are cases in which the introduction of a proper (i.e., non-degenerate) prior distribution will make a parameter that is otherwise non-identified in the frequentist case, identified in the Bayesian case. Moreover, Florens & Simoni (2021) demonstrate via latent variable models, that Markov chain Monte Carlo algorithms in fact show better mixing properties when parameters are not identified than when placing identification constraints on a model, as is typically done to achieve identification in the frequentist domain.

For this article, we follow the discussion in Palomo et al. (2007). Specifically, they consider the problem of Bayesian identification as one of Bayesian learning—namely whether the estimates from the posterior distribution differ from those of the prior distribution when having encountered the data. This implies that Bayesian identification is tied closely to the specification of the prior distribution. As an example given in Palomo et al. (2007), certain parameters such as variances require *positivity* constraints because variances cannot be negative. Thus to ensure Bayesian identifiability of the variances of a model, certain distributions such as the half-normal or half-Cauchy may be employed.

It should be noted that, in principle, Bayesian methods could allow one to choose informative prior distributions that obtain sensible posterior estimates which would otherwise not be identified in a frequentist framework. As such, Palomo et al. (2007), in contrast to Florens & Simoni (2021), prefer setting frequentist identification constraints within Bayesian models to reduce the dependence on prior distributions for those parameters.

For this article, we will be implementing a Bayesian structural equation modeling (SEM) algorithm to estimate our growth models. The SEM approach to growth modeling was originally considered by Muthen (1991, see also; Willett & Sayer, 1994) and further developed in Bollen & Curran (2006) and Grimm et al. (2017). Within the SEM framework, the growth parameters are considered latent variables obtained by setting constraints in the factor loading matrix such that the intercepts and growth rates are essentially factor scores (random effects). A mean structure is added so that the average intercept and average slope are estimated as factor means (see Bollen & Curran, 2006; Kaplan, 2009; Willett & Sayer, 1994, for further discussion). Thus, we follow the suggestion in Palomo et al. (2007), noting that the constraints that are placed on growth curve models to render estimates of the starting point and pace of progress virtually guarantees model identification. Nevertheless, we carefully assess convergence of the MCMC algorithm, described next.

### 3.2 Model estimation

The development of *Markov chain Monte Carlo* sampling methods such as the Metropolis-Hastings (M-H) and Gibbs sampling algorithms and their implementation in Bayesian software programs have made it possible to bring Bayesian statistics into mainstream practice (Gilks et al., 1996). However, these two algorithms suffer from a severe practical limitation—namely, as the number of parameters increases, the number of directions that the algorithm can search increases exponentially while the M-H acceptance probability decreases. Thus, these two algorithms can take an unacceptably long time to converge to the posterior distribution, resulting in a highly inefficient use of computer resources (Hoffman & Gelman, 2014).

#### 3.3 Hamiltonian Monte Carlo

An approach for addressing the problem of computational inefficiency has emerged from the development of *Hamiltonian Monte Carlo*. The mathematics behind HMC arises from the field of Hamiltonian dynamics which was designed to address problems in quantum chromodynamics in the context of the orbital dynamics of fundamental particles. Hamiltonian Monte Carlo underlies the Stan programming environment, which we will be using for the example in this article.

Following closely the discussion given in Kaplan (2023) and drawing on excellent intuitive introductions to HMC by Betancourt (2018, 2019) the problem associated with the inefficient use of computer resources when implementing M-H or Gibbs algorithms is a result of the geometry of probability distributions when the number of parameters increases. In particular, although the density of a distribution is largest in the neighborhood near the mode, the volume of that neighborhood decreases and thus has an inconsequential impact on the calculation of expectations. At the same time, as the number of parameters increases, the region far away from the mode has greater volume but much smaller density and thus also contributes negligibly to the calculation of expectations. The neighborhood between these extremes is called the *typical set*, which is a subspace of the support of the distribution. This “Goldilocks zone” represents a region where the volume and density are just right, and where the mass is sufficient to produce reasonable expectations. Again, outside of the typical set, the contribution to the calculation of expectations is inconsequential and thus a waste of computing resources (Betancourt, 2018).

The difficulty with the M-H and Gibbs algorithms is that although they will eventually explore the typical set of a distribution, it might be so slow that computer resources will be expended. This problem is due to the random walk nature of these algorithms. For example, in the ideal situation for a small number of parameters, the proposal distribution of the M-H algorithm (usually a Gaussian proposal distribution) will be biased toward the tails of the distribution where the volume is high while the algorithm will reject proposal values if the density is small. This will push the M-H algorithm toward the typical set as desired. However, as the number of parameters increase, the volume outside the typical set will dominate the volume inside the typical set and thus the Markov chain will mostly end up outside the typical set yielding proposals with low probabilities and hence more rejections by the algorithm. This results in the Markov chain getting stuck outside the typical set and thus moving very slowly, as is often observed when employing M-H in practice. The same problem just described holds for the Gibbs sampler as well.

The solution to the problem of Markov chains getting stuck outside the typical set is to come up with an approach that is capable of making large jumps across regions of the typical set, such that the typical set is fully explored without the algorithm jumping outside. This is the goal of HMC. Specifically, HMC exploits the geometry of the typical set and constructs transitions that “…glide across the typical set toward new, unexplored neighborhoods” (Betancourt, 2018, p. 18). To accomplish this controlled sojourn across the typical set, HMC exploits the correspondence between probabilistic systems and physical systems. As discussed in Betancourt (2018), the physical analogy is one of placing a satellite in a stable orbit around Earth. A balance must be struck between the momentum of the satellite and the gravity of Earth. Too much momentum and the satellite will fly off into space. Too little, and the satellite will crash into Earth. Thus, the key to gliding across the typical set is to carefully choose an auxiliary momentum parameter to the probabilistic system. This momentum parameter is essentially a first-order gradient calculated from the log-posterior distribution.

#### 3.4 No-U-turn sampler (NUTS)

Hamiltonian Monte Carlo yields a much more efficient exploration of the posterior distribution compared to random-walk M-H and Gibbs. However, HMC requires user-specified parameters that can still result in a degree of computational inefficiency. These parameters are referred to as the step size 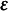 and the number of so-called *leapfrog* steps *L*. If 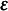 is too large, then the acceptance rates will be too low. On the other hand, if 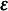 is too small, then computation time is being wasted because the algorithm is taking unnecessarily small steps. With regard to the leapfrog steps, if *L* is too small, then the draws will be too close to each other, resulting in random walk behavior and slow mixing of the chains. If *L* is too large, then computational resources will be wasted because the algorithm will loop back and repeat its steps (Hoffman & Gelman, 2014). Although 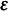 can be adjusted “on the fly” through the use of adaptive MCMC, deciding on the appropriate value of *L* is more difficult, and a poor choice of either parameter can lead to serious computational inefficiency. To solve these problems, the *NUTS* algorithm was developed by Hoffman & Gelman (2014), which is designed to mimic the dynamics of HMC, while not requiring the user to specify 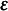 or *L*. The NUTS algorithm is implemented in Stan (Stan Development Team, 2021).

## 4 Bayesian probabilistic prediction

In the previous section, we discussed the specification, identification, and estimation of the growth curve modeling framework that we will use in our example. Again, our goal is to optimize the prediction of the pace at which countries are trending toward or away from the SDGs for the purposes of accurately predicting future outcomes of country-level mathematics competencies, and thus, the focus of this article is on prediction in the longitudinal context. We argue that a central characteristic of statistics is to develop accurate predictive models, and, all other things being equal, a given model is to be preferred over other competing models if it provides better predictions of what actually occurred (Dawid, 1984). Indeed, it is hard to feel confident about inferences drawn from a model that does a poor job of predicting the extant data. For our problem, the question is how to develop accurate predictive models of country-level pace, and, importantly, how to evaluate the accuracy of the predictions. Only then may we feel comfortable using optimal predictions of pace to predict future observable outcomes. We argue that the evaluation of Bayesian predictive models is best situated in the context of *Bayesian decision theory*.

Bayesian decision theory (see e.g. Berger, 2013; Good, 1952; Lindley, 1991) provides a natural and intuitive approach to evaluating Bayesian predictive models. Specifically, as will be expanded on below, Bayesian decision theory casts the problem of predictive evaluation in the context of minimizing *expected loss*—that is, the penalty that is accrued from using a particular model to predict future observations. The less the expected loss, the better the model is at predictive performance in comparison to other models.

### 4.1 Fixing notation and concepts

Following closely the review in Kaplan (2021) but modified to focus on the prediction of the pace of progress, let 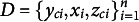 be a set of data assumed to be fixed in the Bayesian sense^4^, where 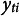 is the outcome of interest at cycle *t* for country *i*, 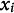 is a (possibly vector-valued) set of time-invariant predictors, and 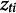 is a set of time varying-predictors for country *i*. Further, let ( 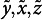 ) be a future observation of the outcome of interest and the set of predictors, respectively. Finally, let 
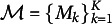
 represent a set of individual models specified to provide predictions of the pace of progress 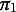 and let 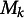 represent a specific model for the pace of progress. Each 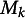 will eventually be a member in the ensemble 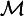 .

The elements of Bayesian decision theory that we adopt in this article have been described by Bernardo & Smith (2000) and Vehtari & Ojanen (2012) among many others. These elements consist of (a) an unknown state of the world denoted as 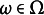 , (b) an action 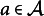 , where 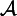 is the action space, (c) a loss function 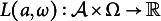 that rewards an action *a* when the state of the world is realized as 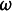 , and (d) 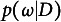 representing one’s current belief about the state of world conditional on observing the data, *D*.

To provide a context for these ideas, and in anticipation of our empirical example, consider the problem of predicting a future outcome of mathematics proficiency for PISA participating countries. In line with Bernardo & Smith (2000), Lindley (1991), Vehtari & Ojanen (2012) and Berger (2013) and the notation given previously for country *i* at time *t*, (a) the states of the world correspond to the future mathematics proficiency scores from future cycles of PISA, that is, 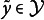 , (b) the action 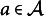 is the actual prediction of those future observations based on using an optimized prediction of the pace of progress 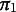 , (c) the loss function 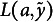 defines the loss attached to the prediction, and (d) a posterior predictive distribution, 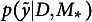 , that encodes our belief about the pace of progress in mathematics proficiency conditional on the data, *D*.

### 4.2 Loss functions for evaluating predictions

The goal of predictive modeling is to minimize the loss associated with taking an action *a* among a set of actions in the action space 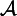 . A number of loss functions exist, but common loss functions rest on the negative *quadratic loss* function 
(6)

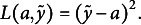

The optimal action 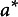 is the one that minimizes the *posterior expected loss*, written as 
(7)

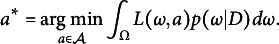

The idea here is to take an action *a* that minimizes the loss *L* when the future observation is 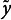 . Clyde & Iversen (2013) show that the optimal decision obtains when 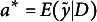 , which is the posterior predictive mean of 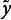 given the data *D*. Under the assumption that the true model exists and is among the set of models under consideration, this can be expressed as 
(8)

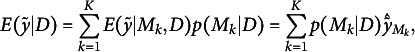

where 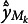 is the posterior predictive mean of 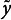 under 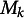 and 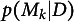 is the *posterior model probability* (PMP) associated with model *k*. The PMP can be expressed as 
(9)

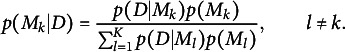

where 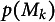 the prior probability for model *k*. The typical default uniform prior mass over the model space and non-informative priors for the parameters of each model, but software programs such as BMS (Zeugner & Feldkircher, 2015) allows for other prior choices over both the model space and the parameters. Equations (8) and (9) define BMA.

It is important to note that when considering the selection of a single model, one might be tempted to choose the model with the highest PMP. In the case of only two models, the model with the largest PMP will be the closest to the BMA solution. However, for more than two models, Clyde & Iversen (2013) point out that the model closest to the BMA solution might not be the one with the largest PMP.

### 4.3 Scoring rules for probabilistic prediction

A critical part of building ensemble prediction models is to have a method for assessing the quality of an ensemble’s predictive performance, sometimes referred to as a model’s predictive *skill*. Popular methods used in economic forecasting and weather forecasting, among other areas, for assessing predictive skill are referred to as *scoring rules* (see, e.g., Bernardo & Smith, 2000; Gneiting & Raftery, 2007; Jose et al., 2008; Merkle & Steyvers, 2013; Winkler, 1996). Scoring rules provide a measure of the accuracy of probabilistic predictions, and a prediction can be said to be “well-calibrated” if the assigned probability of the outcome matches the actual proportion of times that the outcome occurred (Dawid, 1982). For this article, we focus on one strictly proper scoring rule that is commonly used to evaluate predictive accuracy—namely, the *Kullback–Leibler Divergence* score (Kullback & Leibler, 1951; Kullback, 1959, 1987).

#### 4.4 Kullback–Leibler divergence score

Consider two distributions, 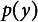 and 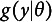 , where 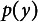 could be the distribution of observed mathematics proficiency scores, and 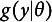 could be the prediction of these mathematics scores based on a model. The KLD between these two distributions can be written as 
(10)

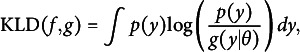

where 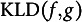 is the information lost when 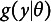 is used to approximate 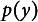 . For example, the actual mathematics outcome scores might be compared to the predicted outcome using BMA along with different choices of model and parameter priors. The model with the lowest KLD measure is deemed best in the sense that the information lost when approximating the actual mathematics outcome distribution with the distribution predicted on the basis of the model is lowest.

## The tripartite 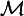 -framework

5

We noted earlier that BMA rests on a very restrictive assumption, namely that there is a true (or correct) model for predicting the pace of progress, denoted as 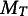 , and that this true model for the pace of progress 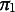 is in the set of models that is being averaged. If this assumption does not hold, then conventional BMA does not makes sense because the priors on the model space are elicited to reflect the analyst’s belief about the existence of the true model within the full set of models under consideration. Nevertheless, if we assume that 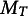 is in the space of models under consideration, this is referred to as the 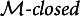 framework, introduced as one of three modeling frameworks ( 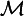 -frameworks) by Bernardo & Smith (2000) and further discussed in Clyde & Iversen (2013).

The 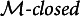 framework for BMA may be especially difficult to justify in the social and behavioral sciences. However, as pointed out by Bernardo & Smith (2000), there may be cases in which it is reasonable to act as though there is a true model. For example, we may wish to act as though 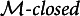 holds when a model has demonstrated good predictive capabilities under a wide variety of situations, but that under a new situation, new uncertainties arise. Such justification might be reasonable in cross-sectional studies, but may be particularly difficult in longitudinal studies where information from previous longitudinal studies may be hard to come by. Still, as long as the analyst is comfortable assigning model priors, then the 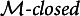 framework can be adopted. Nevertheless, the truth or falsity of the 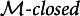 framework notwithstanding, it is important to reiterate that conventional BMA takes place under the 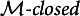 framework and, indeed, readily available BMA software typically employ a non-informative prior to the space of models as a default, with the idea that the true model lies in the model space.

### The 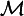 - *complete framework*

5.1

With the 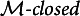 assumption unlikely to hold in practice, we are faced with the problem of how to obtain the benefits of model averaging with respect to predictive accuracy. One approach would be to create a list of simpler “proxy” linear models, 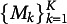 specified for clarity of communication and ease of analysis (Bernardo & Smith, 2000). Each of these models would be evaluated in light of the true model. This is referred to as the 
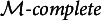
 framework (Bernardo & Smith, 2000). Under 
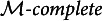
 , BMA would not, in principle, be conducted as it does not make sense to place a discrete prior on the model space when one does not believe that 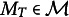 . Instead, as suggested by Clyde & Iversen (2013), Yao et al. (2018), and Vehtari & Ojanen (2012) one simply selects the model 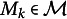 that minimizes expected loss with respect to predictive distributions. However, this suggests that a single model is being used for predictive purposes with the result that model uncertainty is still not being addressed.

### The 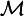 -*open framework*

5.2

If it is difficult to justify model priors as required under 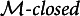 , and if selecting a single model under 
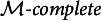
 that minimizes expected loss is not satisfactory, then we need an approach that allows for model averaging without the need to assume 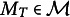 . This is referred to as the 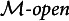 framework (Bernardo & Smith, 2000). An example of an 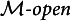 problem is in specifying a set of regression models with different choices of predictors. These different regression models would represent reasonable alternative belief models, and so using posterior model probabilities as weights, each model would yield a separate score without presuming the existence of a true model underlying any of the separate models. These models would be combined using their scores as weights, and the resulting predictive distribution would be obtained. This type of model averaging in the 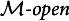 framework describes the methodology of *Bayesian stacking* which we consider next.

## 6 Ensemble prediction using Bayesian stacking

The method of *stacking* was originally developed in the machine learning literature by Wolpert (1992) and Breiman (1996) and brought into the Bayesian paradigm by Clyde & Iversen (2013). A review of Bayesian stacking applied to large-scale educational assessments can be found in Kaplan (2021) and extensions of Bayesian stacking applied to multilevel models can be found in Huang & Kaplan (2024). The basic idea behind stacking is to enumerate a set of 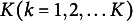 models and then create a weighted combination of their predictions.

In what follows, we describe the process of ensemble modeling via Bayesian stacking for the pace of progress parameter, 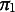 noting that the same approach was used for the starting point, 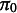 . Returning to our example, we can specify a set of *ensemble member* models of the pace of progress in mathematics proficiency as 
(11)

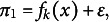

were 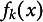 are different models for the pace of progress 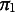 conditional on a vector of predictors of the pace—e.g., some models may include only demographic predictors, others may include various combinations of attitudes and behaviors related to mathematics, and still others may be highly complex functional forms for the prediction of rates of change. To begin, define a set of weights as a simplex, 
(12)

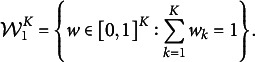

The stacking problem can be written in terms of either minimizing the divergence *d* as 
(13)

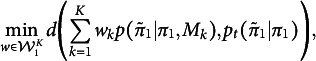

or maximizing the log score 
(14)

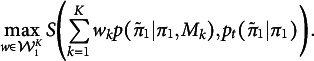

To approximate the full predictive distribution 
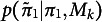
 Yao et al. (2018) use the leave-one-out (LOO) predictive distribution 
(15)

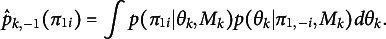

The stacking weights using the log score are the solution to 
(16)

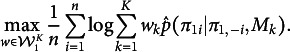

Optimization of Equation 16 uses algorithms in Stan (Stan Development Team, 2021). Inspection of Equations (15) and (16) reveal that the the LOO predictive density is being used twice and so could leave to overally optimistic conclusions. To remedy this, a full Bayesian approach referred to as Bayesian hierarchical stacking (BHS) could be used, but was not implemented in this article (see Huang & Kaplan, 2024, for an application of BHS to large-scale assessments).

### 6.1 LOO cross-validation

We see from Equation (16) that a method is needed to estimate 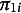 based on 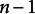 observations leaving the *i*th country out, and the most common approach is referred to as *leave-one-out cross validation*. LOO-cross-validation (LOO-CV) is a special case of *q*-fold cross-validation (*q*-fold CV) when 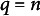 . In *q*-fold CV, a sample is split into *q* groups (folds) and each fold is taken to be the validation set with the remaining 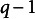 folds serving as the training set. For LOO-CV, each observation serves as the validation set with the remaining 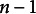 observations serving as the training set. Leave-one-out cross-validation is available in the R software program loo (Vehtari et al., 2019).^5^

Following Vehtari et al. (2017), let 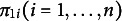 be an *n*-dimensional vector of pace parameters following a distribution conditional on parameters 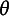 - viz. 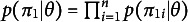 . Given a prior distribution on the parameters, 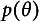 , we can obtain the posterior distribution, 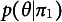 as well as a posterior predictive distribution of predicted values 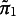 written as 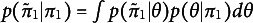 . The Bayesian LOO-CV rests on the derivation of the *expected log point-wise predictive ity* (ELPD) for new data defined as 
(17)

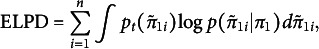

where 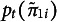 represents the distribution of the true but unknown data-generating process for each country’s pace of progress 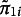 and where Equation (17) is approximated by cross-validation procedures. The ELPD provides a measure of predictive accuracy for the *n* data points taken one at a time (Vehtari et al., 2017). From here, the Bayesian LOO estimate can be written as 
(18)

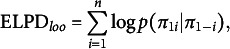

where 
(19)

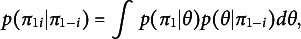

which is the LOO predictive distribution using the log predictive score to assess predictive accuracy. It is useful to note that an information criterion based on LOO (LOO-IC) can be easily derived as 
(20)

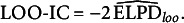

which places the LOO-IC on the “deviance scale” (see Vehtari et al., 2017 for more details on the implementation of the LOO-IC in loo). Among a set of competing models, the one with the smallest LOO-IC is considered best from an out-of-sample point-wise predictive point of view.

As pointed out by Vehtari et al. (2017), it can be time-consuming to calculate exact LOO-CV and this may be a reason why LOO-CV is not widely adopted. To remedy this, Vehtari et al. (2017) developed a fast and stable approach to obtaining LOO-CV referred to as *Pareto-smoothed importance sampling* (PSIS-LOO) (see Vehtari et al., 2017, for more details). The PSIS approach is implemented in loo (Vehtari et al., 2019).

### 6.2 Other types of stacking weights

In addition to stacking weights based on the ELPD, we will also examine the performance of two alternative stacking weights: *pseudo-BMA* (PBMA) and *pseudo-BMA+* weights.

#### 6.3 Pseudo-BMA weights

Pseudo-BMA (PBMA) weights were proposed by (Geisser & Eddy, 1979, see also; Gelfand, 1996; Yao et al., 2018). The basic idea behind PBMA is as follows. First, as discussed in Yao et al. (2021)), LOO-CV has connections to other types of weights that can be used for stacking. For example, in the case of maximum likelihood estimation, LOO-CV weights are asymptotically equivalent to Akaike information criterion (AIC) weights (Akaike, 1973) that are used in frequentist model averaging applications (see also; Burnham & Anderson, 2002; Fletcher, 2018; Yao et al., 2018). As a method of model selection, earlier work by Geisser & Eddy (1979), see also; Gelfand, 1996) criticised the underpinnings of Bayes factors and suggested substituting the marginal likelihood of the *k*th model, 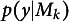 , used in the calculation of Bayes factors with Bayesian leave-one-out cross-validation predictive densities, defined as 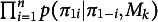 . Yao et al. (2018) refer to AIC weighting using LOO-CV predictive densities as pseudo-BMA weighting.

#### 6.4 Pseudo-BMA+ weights

The difficulty with PBMA weights is that they do not take into account uncertainty in the LOO estimation of the weights. To address this Yao et al. (2018) proposed an approach that combines the Bayesian bootstrap (see Rubin, 1981) with the ELPD defined earlier. They refer to this approach as *pseudo-BMA+* (PBMA+). Following Yao et al. (2018), the essential idea behind PBMA+ is that the posterior distribution of the realizations of a random variable *Z*, that is 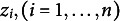 , follows a Dirichlet(1,…,1) distribution—i.e., a uniform distribution. Taking samples from this distribution yields Bayesian bootstrap samples from which parameters from this distribution can be calculated. Specifically, let 
(21)

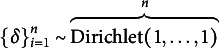

be a set of posterior probabilities for all 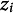 representing one Bayesian bootstrap replication. From here, a parameter of interest represented as a function of *Z*, 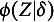 can be obtained as 
(22)

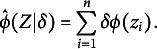

Repeated sampling from 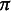 then results in an estimate of 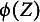 .

With regard to stacking, Yao et al. (2018) note that the ELPD based on LOO can be highly skewed and argue that the Bayesian bootstrap might be an improvement over the usual Gaussian approximation. The PBMA+ weighting follows essentially the same line of argument as the conventional Bayesian bootstrap. That is, define for each model *k*, 
(23)

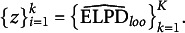

Taking *B* bootstrap samples 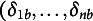 ), 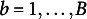 from 
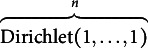
 allows us to calculate the weighted means as 
(24)

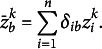

From here, a Bayesian bootstrap sample of the stacking weight for model *k* based on bootstrap samples of size *B* can be obtained as 
(25)

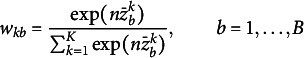

leading to the final PBMA+ weight for model *k*

(26)

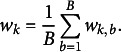

Of importance to this article, Yao et al. (2018) showed that PBMA+ performs better than BMA and PBMA in 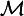 -open settings, but not as well as stacking using the log score. This article adds to the existing literature by comparing stacking based on the ELPD 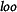 to PBMA and PBMA+ weights in the context of growth curve models applied to large-scale assessments.

## 7 Example: Stacking growth curve models of PISA mathematics proficiency

This article will apply Bayesian stacking to data from 53 countries that have participated in PISA from 2009 to 2022. Launched in 2000 by the OECD, PISA is a triennial international survey that aims to evaluate education systems worldwide by testing the skills and knowledge of 15-year-old students and is, arguably, the most important policy-relevant international survey currently operating. In 2022, 690,000 15-year-old students attending educational institutions in lower secondary education grades or higher from 81 countries and economies took an internationally agreed-upon two-hour test (OECD, 2023b). Students were assessed in reading, mathematics, science, collaborative problem solving and financial literacy. Available country-level results already account for the complex sampling design and plausible value methodology for obtaining mathematics literacy scores. A detailed account of the PISA design can be found in the overview by Kaplan & Kuger (2016).

We will focus on country-level longitudinal outcomes in mathematics proficiency using data from PISA 2009 to PISA 2022. Although longer time points are available, it was decided to only include countries with complete data on the mathematics outcome so that predictive analyses were not dependent on imputation of missing data. The list of these countries is shown in Table 1.

**Table 1:** Fifty-four countries and economies with complete mathematics assessment data from 2009 to 2022


Albania	Argentina	Australia	Austria	Belgium
Brazil	Bulgaria	Canada	Chile	Chinese Taipei
Colombia	Croatia	Czech Republic	Denmark	Estonia
Finland	France	Germany	Greece	Hong Kong-China
Hungary	Iceland	Indonesia	Ireland	Israel
Italy	Japan	Jordan	Kazakhstan	Korea
Latvia	Lithuania	Macao-China	Mexico	Montenegro
The Netherlands	New Zealand	Norway	Peru	Poland
Portugal	Qatar	Romania	Singapore	Slovak Republic
Slovenia	Spain	Sweden	Switzerland	Thailand
Turkey	United Kingdom	United States	Uruguay	

*Note*: Luxembourg did not have data for PISA 2022 and the Russian Federation was excluded from the PISA 2022 assessment due to the war in Ukraine.

## 8 Proposed workflow

For this article, we use the R program blavaan, which is a lavaan-type SEM interface to rstan (Merkle et al., 2021; Rosseel, 2012; Stan Development Team, 2023) for the estimation of the starting point and pace of progress. The program mice (van Buuren & Groothuis-Oudshoorn, 2011) was used for predictive mean matching imputation of missing data. Weight calculations under EPLD 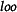 , PBMA, and PBMA+ used the program loo (Vehtari et al., 2019), and model evaluation used LaplacesDemon (Statisticat & LLC., 2021). The full analysis with eight models, including growth curve modeling, missing data imputation, weight calculation, model evaluation, and knitted with rmarkdown (Allaire et al., 2024), took forty-five minutes using twenty cores on a Dell laptop. The code for this analysis is available at https://bise.wceruw.org/publications.html.

The workflow for our example is as follows.

- **Step 1**: Estimate a latent growth curve model without predictors. Different growth curve models should be specified and compared via LOO-CV.
  - **Step 1a**: If each model gives rise to very different growth rates, it may be useful to perform Bayesian stacking on these models first. The stacked predictive distributions could then be used below in a stacking that involves models with different predictors. We refer to this as *super-ensemble* modeling.
- **Step 2**: Create a list of *ensemble members*, each one being a substantively distinct model predicting the growth rate. For each member model, estimate the pace of progress from Equation (1) and compare models based on the ELPD 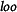 values, and, relatedly the LOO-IC. Compare ELPD 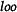 weights to PBMA and PBMA+ weights. In practice, the analyst would likely choose just one of these weights, but there is no harm in comparing outcomes using the other weights.
  - **2a**: (Optional, but recommended): Here, one could check on the relative distinctiveness of the individual member models via posterior predictive checking of each model separately (see Nold et al., 2024; Yao et al., 2021).^6^
- **Step 3**: Combine the predictive distributions of the growth rates weighted by the stacking weights. The code we use for stacking predictive distributions is available at (**blinded for review**)
- **Step 4**: Evaluate the stacked predictive distribution of linear growth against the linear growth rate in Step 1 via the KLD scoring rule in Equation (10). In theory, the stacked predictive distribution should have a smaller KLD than that of any of the model members.
- **Step 5**: Based on the average pace of progress from the stacked predictive distribution, produce prediction plots.
  - **Step 5a** (Optional but recommended): For this step, pseudo out-of-sample predictions may be desirable. For pseudo out-of-sample prediction, the stacked pace of progress is estimated on all but the *T*th-1 time point. Then, with the stacked pace of progress in hand, one simply estimates the proficiency scores for the *T*th time point and compares the predictions to the actual values at time *T* using KLD. One can also compare the different weight methods described above, and the weighting method that yields the lowest KLD would be chosen for the prediction task at hand. Once the analyst has settled on a weighting method that showed the best performance using the KLD, then the analyst can move on to out-of-sample prediction using the intercept and stacked pace of progress.

## 8 Results

**Figure 1:**
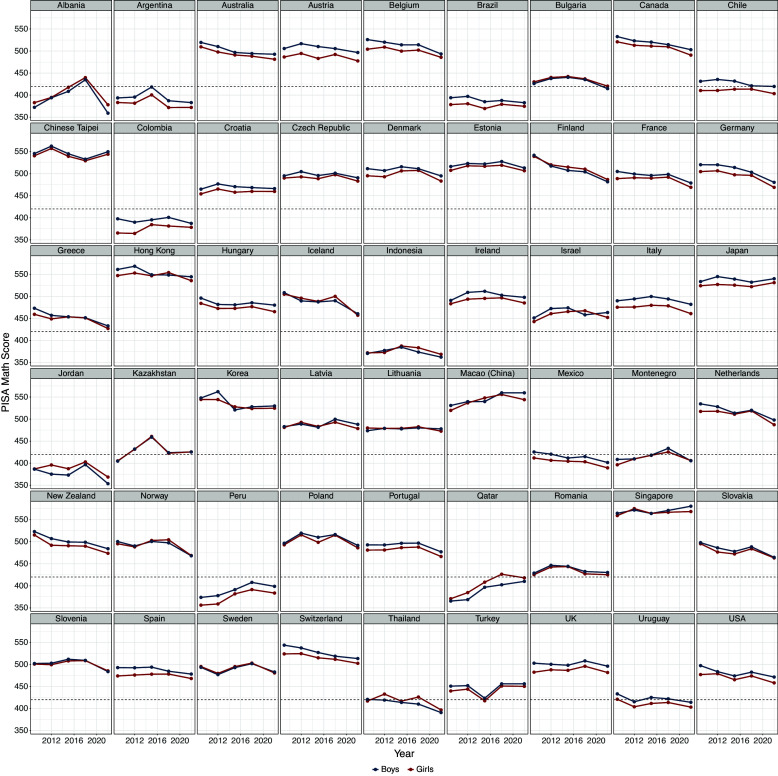
Trend lines for PISA mathematics proficiency from 2009 to 2022. The red line is for the girls, the blue line is for boys, and the horizontal black line is the cutoff for PISA level-2 minimum proficiency. Note that Chinese–Taipei is shown here but was removed from the stacking analysis due to a large amount of missing data on relevant predictors.

To begin, Figure 1 displays the observed trajectories from 2009 to 2022 for mathematics proficiency across the countries for this analysis, along with the OECD international average. The red line represents the trajectories for girls and the blue line represents the trajectories for boys. The black horizontal line represents the PISA Level-2 proficiency cutoff (OECD, 2024). Countries below the black line are performing, on average below minimum proficiency in mathematics.

We observe a fair amount of variation in mathematics proficiency over time, as well as a noticeable decline for many countries (including the overall international average) from 2018 to 2022. For example, Finland had been showing a steady decline in mathematics proficiency from 2009, with perhaps a slightly steeper decline in 2022. Alternatively, Albania was showing steady improvement in mathematics (equivalently for boys and girls), but a sudden drop off to slightly below the minimum proficiency level in 2022. Finally, Chinese Taipei shows relatively stable performance in mathematics through 2022. Thus, to reiterate, the purpose of this article is (1) to estimate the country-level starting point and pace of progress in mathematics proficiency over time, (2) create a set of member models to explain variation in these parameters over countries, (3) stack the predictive distributions derived from the member models to obtain an ensemble distribution of these parameters, and (4) use the starting point and pace of progress means of the ensemble distribution to predict future mathematics proficiency.

### 8.1 Single model members

For Step 1 of our workflow, we begin with a single model approach in which we examine several models with slight differences in the estimation of the pace of progress. This approach is akin to so-called *single model ensemble forecasting* found in the weather forecasting literature (Dutton, 2021). The following is the list of member models for estimating the pace of progress in PISA mathematics proficiency.

1. **Linear growth curve model**. This model was described in Equation (1). The basis terms for this model range from the 2009 to 2022 cycles of PISA and are fixed and coded as 0,3,6,9,13. Note that the last basis term for 2022 is 4 years after the latent basis term for 2018 reflecting the delay of the 2022 PISA cycle due to the pandemic.
2. **Latent Basis Model A**. This model is the same as the linear growth model except that the latent basis term for 2022 is allowed to be freely estimated. This model assumes that the decrease in mathematics achievement scores occurred mainly as a result of the pandemic and shown in the PISA 2022 results.
3. **Latent basis model B**. This model is the same as latent basis model A except that we also allow the latent basis term for 2018, as well as 2022, to be estimated by the data. This model assumes that the decrease in mathematics was already occurring prior to the pandemic and was already manifest in the PISA 2018 scores.

### 8.2 Single model ensemble results

Here and throughout, we base our decisions regarding model predictive quality on models for the pace of progress, 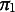 , and not for the starting point, 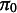 . We discuss the implications of this decision in the Summary and Discussion section.

In Table 2 we present the posterior rates of progress under the linear growth model and two latent basis models. We find that the linear slope model and the latent basis models do not show substantively important differences in predictive quality either overall or for boys and girls separately in terms of the LOO-IC. A possible explanation for this finding is that the across countries the trends are, for the most part, linear, and so relaxing the the strict linear model by allowing for data-based estimation of the basis terms does not contribute much to overall predictive skill. That said, we do find some decline in country-level mathematics scores is greater when allowing a degree of non-linearity induced by the possible impact of the pandemic measured at the PISA 2022 cycle. Moreover, focusing on latent basis M1, we find that, at the country level, boys are declining in mathematics at a rate that is 1.5 times faster than for girls. An inspection of the 90% credible interval for this model also reveals that the overall decline, as well as the decline for boys, does not contain zero, whereas the credible interval for the girls does contain zero. This suggests that the trend in mathematics competencies has been relatively flat for girls from 2009 to 2022, whereas the trend has been steadily decreasing for boys and possibly made worse by the pandemic.

**Table 2:** Posterior estimate of starting points and rates of progress, 90% credible intervals (in parentheses), and predictive evaluation under linear and two latent basis models 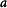

	Overall	Boys	Girls
**Starting point**			
Linear model	470.002 (469.805, 470.202)	470.002 (469.808, 470.198)	470.000 (469.803, 470.197)
Latent basis M1	470.001 (469.814, 470.199)	470.001 (469.805, 470.197)	470.001 (469.805, 470.198)
Latent basis M2	470.008 (469.808, 470.198)	470.000 (469.809, 470.197)	470.001 (469.809, 470.194)
**Pace of progress**			
Linear model	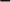 0.503 ( 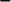 0.977, 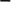 0.040)	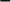 0.654 ( 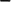 1.097, 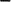 0.227)	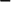 0.405 ( 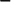 0.917, 0.107)
Latent basis M1	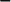 0.564 ( 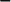 1.071, 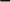 0.019)	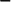 0.700 ( 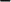 1.097, 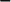 0.227)	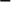 0.457 ( 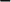 1.047, 0.211)
Latent basis M2	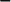 0.641 ( 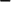 1.234, 0.020)	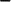 0.772 ( 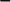 1.284, 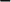 0.158)	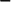 0.556 ( 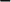 1.233, 0.269)
**Predictive evaluation**			
LOO-IC Linear model	2301.39	2325.35	2305.40
LOO-IC Latent basis M1	2304.13	2328.20	2307.35
LOO-IC Latent basis M2	2304.31	2328.64	2308.30

Having observed in Table (2) that there is no clear distinction among the three models for the estimation of the pace of progress, we focus on obtaining the pace of progress for each country using latent basis M1. Our justification for this model stems from the desire to account for possible non-linearity due to the impact of COVID-19 as reported for PISA 2022. We now move to the next step in our workflow which entails specifying separate and theoretically justified models for the pace of progress.

### 8.3 Proposed multi-model ensemble members

To obtain predictors of the pace of progress in mathematics proficiency, we have drawn on a variety of data sources. Specifically, in addition to the mathematics proficiency outcome, PISA includes measures of school-level resources, accountability, and leadership indicators that can be aggregated to the country level. However, we recognize that caution in interpretation is needed as the meaning of these school-level indicators may change when aggregated to the country level. In addition to PISA, the OECD also provides data on country-level economic indicators such as gross domestic product and government spending on education (see https://data.oecd.org/education.htm). Additional data sources from the OECD were obtained from their annual “Education-at-a-Glance” volumes (e.g., OECD, 2018). Many of the OECD education indicators are also made available to the World Bank through its “EdStats All Indicator Query” system. This system offers more than 4,000 internationally comparable indicators covering different aspects of system-level education. Data are available from the year 1970 onward (see The World Bank, 2019). Finally, UNESCO offers a considerable amount of data in the area of international education. Of relevance to this proposal, UNESCO has already collected selected data linked to the SDGs since 2012 (see UNESCO Institute of Statistics, 2019). UNESCO also has in place a global educational monitoring system for which additional data are readily available (see UNESCO, 2015).

For this article, we have created eight relatively distinct models for the prediction of the pace of progress in mathematics outcomes over the 2009–2022 cycles of PISA using latent basis M1. This approach is akin to so-called *multi-model ensemble forecasting* again found in the weather forecasting domain (Dutton, 2021). Clearly, different models could be specified, but those chosen for this article are based on eight distinct indicator categories based on data sources derived from a consideration of a number of documents and reports from various governmental organizations, in particular, the OECD, UNESCO, and the World Bank. Predictors were chosen and sorted into categories derived from the target definitions of the SDGs (UN General Assembly, 2015) and the theoretical framework of PISA (OECD, 2023a). The predictors were sorted into the categories *SDG 1* or *SDG 4* if they measure aspects that are defined or related to the definitions of the SDG targets 1 or 4, respectively.^7^. Indicators which can be related to aspects of educational quality as defined in the PISA framework were sorted into the category *Instructional quality* for predictors referring to aggregated effects on the school level, or the category *Resources* if they refer to instructional resources on school level. Indicators were sorted into the category *Equity* if they relate to differences between men and women in the selected areas on system level, in most cases a proportion or ratio of men/women, or if the indicators are related to equity in education as defined in the PISA framework. A variable list along with the indicator category and model is given in Appendix. Note also that remaining analyses are based on 53 countries. Chinese-Taipei was removed from remaining analyses due to a large amount of missing data on relevant predictors.

### 8.4 Separate model results

In this section we present the results from separate analyses of the pace of progress, with a focus on each model’s predictive performance based on the expected log predictive density using leave-one-out cross-validation. The results for boys and girls are presented in Table (3).

**Table 3:** Expected log predictive performance based on loo cross-validation for boys (upper panel) and girls (lower panel)

Model	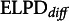	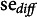	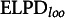	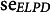	LOO-IC
			*Boys*		
Model 4b	0.000	0.000	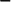 63.071	6.054	126.143
Model 3	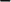 2.093	4.184	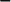 65.165	6.423	130.329
Model 2	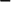 2.796	3.987	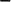 65.868	8.395	131.735
Model 6b	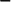 5.499	6.142	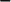 68.571	9.060	137.141
Model 1	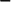 5.584	4.342	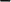 68.655	7.775	137.311
Model 4a	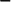 8.640	5.666	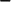 71.711	9.478	143.422
Model 6a	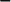 8.658	4.267	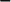 71.730	7.975	143.459
Model 5	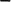 8.711	3.921	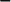 71.783	5.500	143.566
			*Girls*		
Model 4b	0.000	0.000	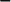 61.113	5.926	122.226
Model 3	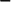 0.955	4.423	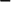 62.068	6.631	124.136
Model 2	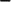 1.123	4.331	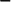 62.236	8.239	124.472
Model 6b	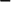 1.638	5.914	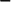 62.751	9.040	125.502
Model 6a	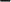 5.836	4.397	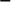 66.949	7.458	133.897
Model 1	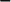 6.154	4.805	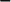 67.267	8.216	134.535
Model 4a	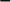 7.992	6.338	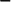 69.105	9.638	138.211
Model 5	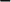 8.795	3.777	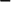 69.908	5.374	139.815

We find for the boys, that Model 4b (resources at the system level) has the lowest LOO-IC. However, on the basis of the 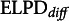 relative to its standard error, Model 1 (size of the education system), Model 2 (SDG goal 4 at the system level), Model 3 (SDG Goal 1 at the system level), Model 4b (resources at the system level), and Model 6b (equity in education) have very similar predictive performance. Regarding the girls, we also find that Model 4b (resources at the system level) has the lowest LOO-IC value and that this model, along with Models 3, 2, and 6b have similar predictive performance.

### 8.5 Stacking results

In Table (4) we present the stacking results for boys and girls. For each model, we present the stacking weights under ELPD 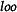 , PBMA, and PBMA+. In addition, we present the KLDs under each of these stacking weights compared to the baseline latent basis model. For comparison purposes, we also show the KLDs for the individual models in comparison to the latent basis model.

**Table 4:** Stacking weights and Kullback–Leibler divergence scores for each model separately and for the ensemble

Model	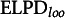	PBMA	PBMA+	KLD
		*Boys*		
Model 1	0.000	0.003	0.017	0.446
Model 2	0.038	0.051	0.142	0.390
Model 3	0.247	0.103	0.219	0.368
Model 4a	0.000	0.000	0.006	0.403
Model 4b	0.487	0.838	0.518	0.331
Model 5	0.000	0.000	0.004	0.356
Model 6a	0.000	0.000	0.001	0.437
Model 6b	0.227	0.003	0.092	0.363
**Ensemble KLD**	**0.316**	**0.323**	**0.323**	
		*Girls*		
Model 1	0.000	0.001	0.006	0.415
Model 2	0.107	0.195	0.183	0.333
Model 3	0.334	0.175	0.223	0.327
Model 4a	0.000	0.000	0.006	0.358
Model 4b	0.058	0.501	0.274	0.300
Model 5	0.000	0.000	0.001	0.340
Model 6a	0.000	0.002	0.005	0.354
Model 6b	0.500	0.124	0.301	0.303
**Ensemble KLD**	**0.279**	**0.274**	**0.284**	

We observe that the KLD scores for each of the weighting methods are uniformly lower than any of the KLDs for the individual models, suggesting that better predictive skill is obtained by using the stacking weights in comparison to any of the member models, including the model with the highest stacking weight. Among the different stacking weights, we find that the KLD associated with the ELPD 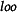 weight is the lowest for the boys, while the PBMA weight is the lowest for the girls but not by very much.

**Figure 2:**
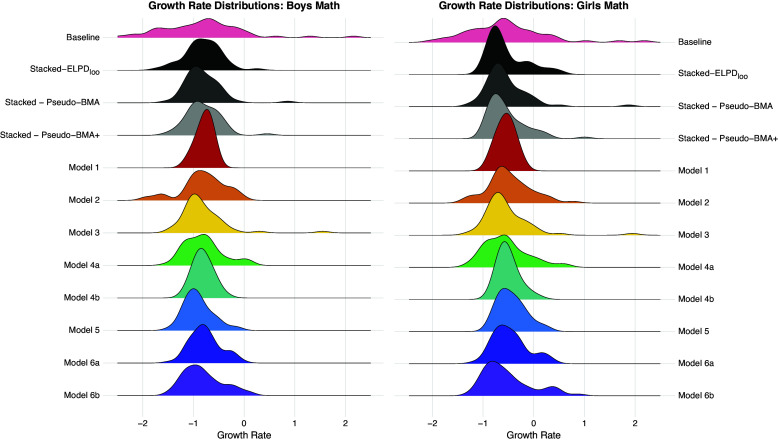
Predictive densities of the pace of progress across different stacking weights and different models for boys and girls.

To gain further insight into stacking, Figure 2 shows the predictive distributions of estimates of the pace of progress across the individual models and the different stacking weights for boys and girls. It appears that the predictive distribution from the ensemble based on ELPD 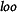 is relatively similar to the distributions from the ensemble member models, and that the baseline distribution exhibits greater density at the lower tail of the distribution than what is captured by the stacked distribution or the model members. We discuss the implications of Figure 2 in the Summary and Discussion section.

### 8.6 Prediction results

**Figure 3:**
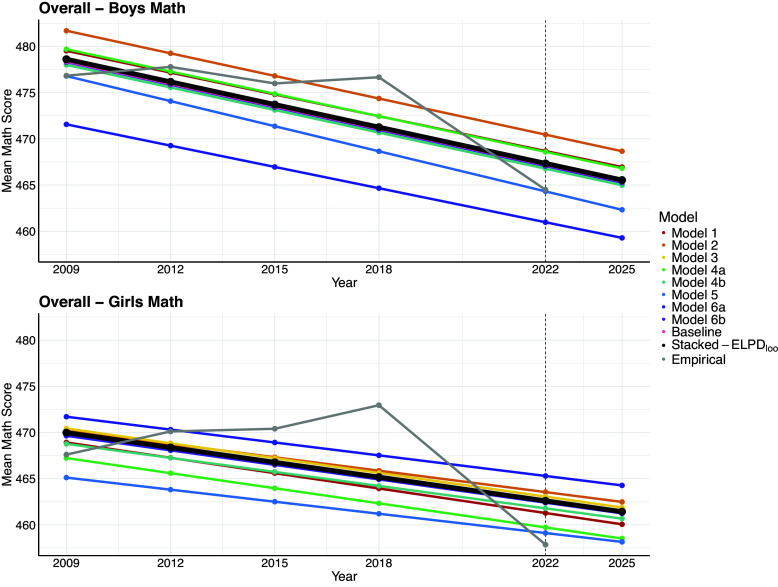
Within-sample and one-cycle ahead predictions for each ensemble member and for the stacked prediction based on ELPD 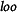 for boys’ and girls’ performance on the PISA mathematics assessment.

In the final step of our workflow we develop predictive plots for mathematical competency. Specifically, with the estimates of the pace of progress based on the ELPD 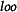 stacking weight in hand, we plot the empirical trajectory of mathematics proficiency scores from 2009 to 2022, and then forecast one period out to 2025—the next PISA cycle. In addition, we show the trajectories based on the posterior estimates of the pace of progress for each ensemble model member. These plots are shown for boys and girls in Figure (3) where we observe that for the boys and the girls, each model predicts a steady decline in mathematics proficiency as measured in PISA. The dark line represents the ensemble prediction of the pace of progress based on the stacking of the linear latent basis model M1. We notice a clustering of models around the ensemble prediction, and this suggests that these models could, individually demonstrate good predictive skill. Nevertheless, from a predictive point of view, the ensemble prediction would, in principle, demonstrate the best predictive skill.

## 9 Summary and discussion

The purpose of this article was to demonstrate an approach to combining predictions of the pace of progress from a set of growth curve models. In line with a long tradition of multi-model inference, we argued that combining predictions from multiple models into an ensemble prediction yields overall better predictive skill than what could be achieved from the selection of a single model.

For this article, we presented a workflow where the first step was to examine predictive skill resulting from relatively minor changes in a growth curve model without adding predictors. We argued that this was similar, though not identical to, single-model ensemble methods found in weather forecasting. In our particular example, we did not find any important differences in the predictive performance of the three changes to the growth models. Had we found important differences, it would have been advisable to use stacking methods to create an ensemble prediction. Not having to create the ensemble, we decided to obtain Bayesian estimates of country-level pace of progress in mathematics proficiency from a latent basis model that allowed for some non-linearity due to the impact of COVID-19.

For the next step of our workflow, we specified a number of different models for the pace of progress based on a collection and merging of data sources containing country-level indicators of education. Six models were specified, and again this phase of our workflow is similar, though not identical to, multi-model ensemble prediction, also found in the weather forecasting domain. As mentioned earlier, we did not judge the predictive quality of the individual models or the ensemble based on models for the starting point. We felt it was more important to stack models for the pace of progress, and it remains a future area of research on how to combine decisions based on related parameters of interest. That said, our results showed that although the boys started off in 2009 with higher mathematics proficiency scores on average across the countries, they have been declining at a noticeably faster rate than girls, which we also observed from the growth rates in Table (2). Of course, gender inequity in the decline in mathematics performance as seen in this article, is unacceptable under any circumstances, but it should also be noted that in real terms, these declines are not very large, resulting, on average, in about two score points every three years. Nevertheless, we argue that the methodologies and workflow provided in this article could be informative to national educational systems as they consider policies to reverse these trends. We hasten to add, however, that although this article provides a promising approach to policy-relevant prediction in longitudinal settings, where obtaining optimal estimates of the pace of progress is the focus, in no way should the results of this specific study be interpreted as informing specific policy decisions.

Although we found Bayesian stacking to provide better predictive performance than any single model in the ensemble, there are open issues with the Bayesian stacking that set the stage for future research. First, the performance of Bayesian stacking is, of course, highly dependent on the set of the member models to be ensembled. We believe that the eight categories of models that we specified are defensible in terms of our review of the extant literature on indicators of global education systems, but naturally other specifications are possible, and we can’t even be certain we have captured functional forms correctly. Nevertheless, the tools available for assessing the quality of predictive models allow for alternative models to be specified and compared in terms of their predictive performance.

Second, even with well developed ensemble members, Bayesian stacking is argued to work well when models are as *distinct* as possible (Breiman, 1996; Clarke, 2003). However, as pointed out by Yao et al. (2021), model distinctiveness is an ideal and there is presently not much guidance on how to quantify distinctiveness among models, or any existing knowledge as to how serious a problem this might be for predictive performance. That said, Figure 2 could provide useful information for assessing the capacity of the ensemble member models to capture broad features of the empirical pace of progress. Specifically, Figure 2 showed that the predictive distributions from the individual models did not seem to capture the lower tail of the baseline empirical pace of progress and this could indicate that other model specifications that capture flatter growth rates should be specified and included in the stack. Using a plot such as Figure 2 to assess the extent to which ensemble member distributions cover the range of possible values of the distribution is beyond the scope of this study but should constitute future research.

Finally, a third area for future research in the context of Bayesian stacking concerns missing data. With regard to this article, we originally considered taking the difference in the predictors from 2022 to 2009 as a measure of their stability over time. However, two important problems emerged. First, for many countries, data on important indicators were simply not reported by the countries. Second, for those that were reported, many were not measured at each time point coincident with the cycles of PISA used in this study (2009–2022). As such, we felt that there were too many missing data points on a number of important indicators and too few countries to begin with to feel comfortable using multiple imputation methods. Thus, we only examined predictors measured in 2009 under the assumption that these predictors have been relatively stable over time. An inspection of some basic descriptive statistics suggests evidence of stability in many, but not all of the predictors. For missing data in 2009, one predictive mean matching imputation (van Buuren & Groothuis-Oudshoorn, 2011) was used.

We recognize that it is better to analyze many (e.g., ¿20) imputed data sets. One approach to analyzing multiply imputed data sets in a Bayesian analysis was proposed by Zhou & Reiter (2010) who recommended analyzing each imputed data set separately and then mixing and summarizing the posterior draws. They find this approach to yield less biased parameter estimates than averaging the parameter estimates. Combining the approach suggested by Zhou & Reiter (2010) along with Bayesian stacking was felt to be beyond the scope of this article, but it does offer an interesting area for future research. Nevertheless, we recognize that our results could change if we had made other decisions regarding missing data.

To conclude, we recognize that the domain of weather forecasting has certain advantages compared to monitoring trends in educational outcomes. In particular, the non-linear dynamics of weather, as well as the near-continuous collection of data, provide rich information on which to build and stack predictions from complex models. Indeed, as noted by the World Climate Service, modern weather ensemble forecasts have been known to have between 12 and 51 model members (Dutton, 2021). Moreover, weather forecasting models are flexible enough to handle exogenous shocks to weather systems, such as the 2024 Icelandic volcano eruption. In the case of monitoring trends in educational outcomes, as we have seen, often simple linear models will suffice, and theory as to why trends in proficiency outcomes have been, in our example, declining for many countries, are not well developed. Still, the methods we are proposing are also flexible enough to handle exogenous shocks to the educational system, such as the disruption to schooling caused by the 2019 global pandemic. It would be interesting to examine whether the workflow that we propose in this article would be applicable to other education targets or even targets associated with different SDGs.

An additional contribution of this article was the demonstration of how ILSAs generally, and PISA in particular, can be leveraged to provide information relevant to tracking progress to the educational sustainable development targets. We believe that this contribution is important because PISA, in particular, has been used by organizations such as the World Bank to monitor and forecast the global impact of the pandemic on educational trends (Azevedo et al., 2020). These endeavors have contributed greatly to our understanding of the impact of the pandemic on schooling. This article adds to the growing literature on monitoring and forecasting educational trends, as well as exogenous shocks to those trends, by combining models that are designed for estimating rates of change, along with Bayesian approaches to optimizing prediction, which we maintain can provide important insights into country-level progress in education.

## Data Availability

The datasets generated during and/or analysed during the current study are available at https://bmer.wceruw.org/publications.html.

## Footnotes

1 Note that we are assuming that the cycles are at fixed intervals and the same for each country, which is reasonable given the operational requirements of PISA.

2 Of course, this assumes that the political system of a country is relatively stable over time.

3 Note that Equations (4) and (5) imply that the same predictors are being used for the starting point and pace of progress, and although that will be the case for this article, it is not necessary, and different predictors for these parameters can be specified.

4 In Bayesian philosophy, conditioning is on observable and fixed data whereas parameters are treated as random variables (Kaplan, 2023).

5 The *widely applicable information criterion* (WAIC) has also been advocated for model selection. Although the WAIC and LOO-CV are asymptotically equivalent (Watanabe, 2010), the implementation of LOO-CV in the loo package is more robust in finite samples with weak priors or influential observations (Vehtari et al., 2017)

6 We discuss the issue of *distinctiveness* in the Summary and Discussion section.

7 SDG 1 “aims to end poverty in all its forms everywhere by the year 2030”; and SDG 4 aims to “Ensure inclusive and equitable quality education and promote lifelong learning opportunities for all” see UN General Assembly (2015)

## Appendix

**Table A1:** Variable names, indicator category, and model number, for multi-model ensemble members

Variable name	Indicator category	Model
% adolescents out of school	Size of education system	M1
Number of students - Primary to post-secondary non-tertiary	Size of education system	M1
Number of students - Tertiary	Size of education system	M1
Children out of school (% of primary school age)	SDG goal 4 on system level	M2
Gross enrolment ratio - primary - both sexes (%)	SDG goal 4 on system level	M2
Gross enrolment ratio - secondary - both sexes (%)	SDG goal 4 on system level	M2
Official entrance age to lower secondary education (years)	SDG goal 4 on system level	M2
Official entrance age to pre-primary education (years)	SDG goal 4 on system level	M2
Official entrance age to primary education (years)	SDG goal 4 on system level	M2
GDP	SDG goal 1 on system level	M3
Gender wage gap at	SDG goal 1 on system level	M3
Poverty rate from age	SDG goal 1 on system level	M3
Annual employment of females as % of employment	SDG goal 1 on system level	M3
Labour force females Annual	SDG goal 1 on system level	M3
Teaching hours - Lower Secondary	Resources on education system level	M4a
Teaching hours - Primary	Resources on education system level	M4a
% GDP expenditure on pre-primary education	Resources on education system level	M4a
% GDP expenditure expenditure on primary education	Resources on education system level	M4a
% GDP expenditure on secondary education	Resources on education system level	M4a
Private spending on education	Resources on education system level	M4a
Index school size	Resources on education system level	M4a
Funding government	Resources on education system level	M4a

*Sources:* OECD, UNESCO, and the World Bank.

Measured in 2010.

Measured in 2011.

**Table A2:**

Variable name	Indicator category	Model
% Rural population	Resources on education system level	M4b
Teaching hours - Early childhood education	Resources on education system level	M4b
Teaching hours - Upper Secondary	Resources on education system level	M4b
Teaching staff compensation pre-primary public (%)	Resources on education system level	M4b
Teaching staff compensation primary public (%)	Resources on education system level	M4b
Teaching staff compensation secondary public (%)	Resources on education system level	M4b
Percentage of teachers in pre-primary education who are female (%)	Instructional quality on school level	M5
Percentage of teachers in primary education who are female (%)	Instructional quality on school level	M5
Percentage of teachers in secondary education who are female (%)	Instructional quality on school level	M5
Part time teach in total 2009	Instructional quality on school level	M5
Trained teachers in primary education (% of total teachers)	Instructional quality on school level	M5
Trained teachers in secondary education (% of total teachers)	Instructional quality on school level	M5
Index minutes per week in mathematics courses	Instructional quality on school level	M5
Number class periods in math	Instructional quality on school level	M5
Index use of ICT at school in general	Instructional quality on school level	M5
Lower secondary school starting age (years)	Equity in education	M6a
Primary school starting age (years)	Equity in education	M6a
Progression to secondary school (%)	Equity in education	M6a
% Repetition rate in lower secondary - both sexes (%)	Equity in education	M6a
Repetition rate in primary education (all grades) - both sexes (%)	Equity in education	M6a
Survival rate to the last grade of primary education - both sexes (%)	Equity in education	M6a
Index highest occupational status of parents	Equity in education	M6b
Index home educational resources	Equity in education	M6b
Index home possessions	Equity in education	M6b
Index ICT - resources	Equity in education	M6b
Index ICT use outside of school for leisure	Equity in education	M6b
Index ICT use outside of school for schoolwork	Equity in education	M6b

*Sources:* OECD, UNESCO, and the World Bank.
